# Childbirth Experience Associated With Maternal and Paternal Stress During the First Year, but Not Child Attachment

**DOI:** 10.3389/fpsyt.2020.562394

**Published:** 2020-09-08

**Authors:** Annaleena Holopainen, Marije L. Verhage, Mirjam Oosterman

**Affiliations:** ^1^ Clinical Child and Family Studies, Faculty of Behavioural and Movement Sciences, Vrije Universiteit Amsterdam, Amsterdam, Netherlands; ^2^ Amsterdam Public Health Research Institute, VU Medical Centre, Amsterdam, Netherlands

**Keywords:** childbirth experience, parental stress, maternal stress, paternal stress, child attachment, postpartum

## Abstract

Women, as well as their partners, can experience childbirth in many different ways. A negative childbirth experience may have adverse effects on the entire family, resulting, for instance, in parental stress symptoms and a weakened parent-child relationship. Parental stress, without sufficient resources to compensate for it, may also in and of itself negatively influence the parent-child relationship. This study contributes to the current knowledge of the psychological effects of childbirth experience by using longitudinal data collected with both self-reports and observational measures, as well as multiple informants (i.e., mothers and partners). The aim of this study was to investigate whether 1) women’s retrospective birth experiences were related to maternal and paternal parenting stress, 2) birth experience was indirectly associated with child attachment via maternal stress, and 3) birth experience was directly related to child attachment. Data were collected from a mixed sample of community and at-risk primipara women (*N* = 1,364), as well as from their partners and children. Retrospective childbirth experience was measured 3 months postpartum with a latent factor consisting of five items asking about the feelings that women have about their childbirth. Parental stress was measured at 3 months postpartum for partners and 3 and 12 months postpartum for mothers using the adult domain of the parental stress index (PSI). Finally, parent-child attachment is observed in a subsample of 223 women and children at 12 months postpartum with the Strange Situation Procedure (SSP). Results show that women’s birth experience was significantly related to both mothers’ and their partners’ parenting stress. However, birth experience was not related to child attachment, neither directly nor indirectly via maternal stress. These findings emphasize the long-lasting impact that childbirth may have on both parents. Future research is still needed to further investigate which protective factors may weaken the association between birth experience and parental stress.

## Introduction

Childbirth may be experienced in many different ways. For most women, giving birth is a positive experience, but it can unfortunately also be experienced as negative, sometimes even as traumatic (1, 2). Negative childbirth experiences not only affect women but also their partners (3–5). Previous research has found that a negative birth experience may result in lower parental well-being and higher (parental) stress symptoms in mothers and partners (4, 6–9). The increased level of parental stress may, in turn, negatively influence the parent-child relationship (10). Some parents may also suffer from severe symptoms of post-traumatic stress disorder after childbirth (11). Accordingly, a traumatic experience may lead to various physiological, emotional, cognitive, and behavioral responses, such as heightened arousal, emotional avoidance, and distancing from others (12). Behavior of this kind may result in insecure child attachment (13), meaning that a traumatic birth experience may also have a direct negative impact on the parent-child relationship. The current study aims to investigate the impact of mother’s childbirth experience on parental stress (i.e., maternal and paternal), the indirect association between negative birth experience and child attachment via maternal stress, and the direct association between negative birth experience and child attachment.

Based on previous research, the prevalence of women’s negative childbirth experiences ranges from 6.8 to 44% (14), and up to a third of women experience childbirth as traumatic (1). Although research including partners is still rather limited, one cross-sectional Swedish study reported that 3% of men experienced their partner’s childbirth as negative (5). For women, a negative or traumatic birth experience may be caused by medical complications, a feeling of not being heard, lack of support, loss of control, or experienced pain (6, 15, 16). Also for men, medical complications and not knowing what is going to happen may lead to experiencing birth as negative or traumatic (4, 5).

Family stress theory (10, 17) states that a family crisis has a reciprocal relationship with the entire family system, meaning that it has consequences on the level of parental stress as well as on the parent-child relationship. The theory states that three factors play a role in determining how much stress is caused by an event. The first factor leading to parental stress is the stressful event itself; in this case, a negative or traumatic birth experience. In family stress theory, stressors can be either normative (e.g., daily hassles, developmental transitions), non-normative (e.g., death of a child, a child’s diagnosis), or chronic stressors (e.g., poverty, a child that requires intensive care) (10). Previous research has identified some external birth-related factors that may increase the chance of experiencing childbirth as negative or traumatic. These are, for instance, emergency cesarean delivery, instrumental delivery, premature birth, stillbirth, or not having one’s partner present when giving birth (1, 18). A negative or traumatic birth experience may thus be either a normative stressor (i.e., developmental transition) or a non-normative stressor (i.e., unexpected and uncontrollable external factor during birth).

The second factor impacting the level of parental stress is the parents’ evaluation of the stressor (10). This includes attitudes, attributions, expectations, definitions, and meanings given to the stressor. Beck (18) already suggested this with the title of his book: Birth trauma lies in the eye of the beholder. This means that a birth that seems rather normal and straightforward to care providers may be experienced as stressful or even traumatic by the woman herself (19).

The third factor influencing parental stress levels in response to an event refers to a combination of personal resources, coping resources, and ability to adapt to a new situation (10). Knowledge of the birthing process or mental health are examples of personal resources, while acceptation of the situation or positive thinking can be seen as adaptive coping strategies. Receiving support, for example, from care professionals or the social network can be seen as both a resource and a coping strategy, which may lead to lower levels of parental stress.

Although a negative or traumatic birth experience may increase the level of parental stress in both women and their partners, (4), the majority of previous research on negative or traumatic birth experiences has focused exclusively on mothers. Previous studies including partners have been either qualitative or have included only small samples [e.g., *N* = 56; (7)]. In order to increase our knowledge on the possible impact of women’s birth experiences on the family system, parental stress in partners should be examined more extensively.

Parental stress is not only problematic in the context of parental well-being, it may also affect parenting behavior and the parent-child relationship (20). Research on parental burnout suggests that parents who experience high levels of parental stress without sufficient resources to compensate for it are at-risk of neglecting their children (20, 21). One explanation for this finding may be that a higher level of parental stress makes a parent less sensitive and responsive to the child’s needs (22, 23). According to attachment theory, low parental sensitivity may lead to insecure or disorganized child attachment (23–25). A child that is insecurely attached does either not show his stress to the caregiver (i.e., insecure-avoidant) or shows angry behavior and is difficult to sooth (i.e., insecure-resistant) (13). Disorganized attachment refers to a situation where a child does not have an organized way to show his stress, in other words, he behaves in a disoriented and contradicting manner (25, 26).

Previous empirical studies have indeed demonstrated an association between higher maternal and paternal stress and insecure child attachment (23, 27). Yet, a study by Adams (28) found that parenting stress was not a strong predictor of parenting behavior or parent-child interaction. Noteworthy is, however, that in this study, parent-child interaction was measured with the Parent-Child Early Relational Assessment [PCERA (29)], which is designed to assess parental sensitivity and therefore differs conceptually from measures that assess parent-child attachment. Previous research on the possible impact of parental stress on child attachment thus remains inconclusive, which is why further research is needed to get a better understanding of this potential association.

Previous research has also been focused on the direct impact of a negative or traumatic birth experience on the parent-child relationship (11, 30–32). Some of the previous studies suggests that a traumatic birth experience, and birth-related postpartum PTSD symptoms, may negatively impact parenting behavior and the parent-child relationship (31, 33, 34), whereas a study by Ayers et al. (11) did not find an association between PTSD symptoms following childbirth and the parent-child relationship. It should be noted that most previous studies used either behavioral codings of parental or child behavior or mothers’ self-reports on their feelings and perceptions of the bond with their child. This is in contrast with the current study in which an observer-rated assessment is used to measure attachment behavior expressed by the child towards the mother.

**Figure 1:**
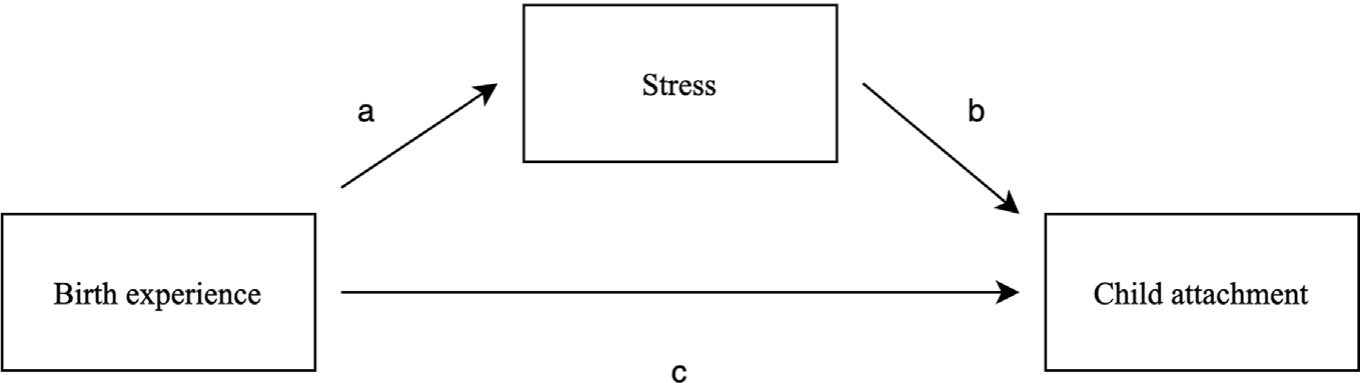
Conceptual model of the study.

In sum, findings on the impact of birth experience and parental stress on the parent-child relationship are inconclusive, and none of the previous studies have used validated, observatory measures to assess child attachment (27, 34). Moreover, most of the previous studies on the effects of birth experience on parental stress and child attachment have a cross-sectional design. Therefore, the current study addresses the outstanding questions on the psychological effects of childbirth experience, more specifically its relationship with parental stress and child attachment, by using longitudinal data and by combining self-reports and observational measures, as well as multiple data sources (i.e., mothers and partners). To the best of our knowledge, this is the first study including all this information (i.e., birth experience, maternal and paternal stress, and child attachment) and testing it in one model, which enables to test both direct and indirect effects. The first objective of this study is to investigate whether women’s retrospective childbirth experiences are related to maternal and paternal parenting stress. A more negative birth experience is hypothesized to be related to a higher level of maternal and paternal stress. The second objective of this study is to investigate whether women’s childbirth experiences are indirectly related to child attachment via maternal stress, and whether women’s childbirth experiences are directly related to child attachment. The following two hypotheses are set regarding this second objective: first, more negative birth experiences are related to insecure child attachment via higher maternal stress, and second, a more negative birth experience is related to an insecure or disorganized child attachment. The conceptual model of the study is presented in **Figure 1**.

## Materials and Methods

### Participants

Data were collected from a mixed sample of community (i.e., ‘large cohort’ and ‘normative group’) and at-risk primipara women (*N* = 1,364), as well as from their partners and children, who participated in the longitudinal Generations^2^ project (www.generaties2.nl/) that takes place in the Netherlands, in the Amsterdam area. The ‘large cohort’ consisted of all participants in the study (i.e., including participants who took part in the questionnaire assessments only), women in the ‘normative group’ who took part in the more extensive measurements (e.g., child attachment), and women in the ‘at-risk’ group who also took part in these more extensive measurements, and had additionally reported experiences with youth care, psychologists, or psychiatrists before the age of 18. A prenatal diagnosis for a congenital abnormality of the fetus was an exclusion criterion for all three groups. For the second objective of this study, we combined the data of the ‘normative’ and ‘at-risk’ subgroup of women and children from whom the attachment data was collected (*N* = 223). This was done to obtain a larger and more representative sample regarding both demographics and attachment outcomes. Later in the text we refer to these subgroups as a ‘focus sample’ and to the entire group as a ‘cohort sample’. In the cohort sample, women were on average 29.5 years old (range 15–43 years, SD = 4.6). The majority of them were born in the Netherlands (91.3%), were either married or cohabiting (90%), and had completed a tertiary education (88.3%). Of the children, 49.2% were boys and 50.7% girls. For two children, gender was not reported. Descriptive information of both samples are presented in **Table 1**.

**Table 1:** Descriptive information of the two samples.

	Cohort sample (N = 1364)	Focus sample (N = 223)
Age of the mother	29.5 years (range 15–43; SD 4.6)	27.9 years (range 15–42; SD 5.9)
Highest education	Primary education 1.2% Secondary education 9.4% Tertiary education 88.3%	Primary education 4.3% Secondary education 17.4% Tertiary education 78.2%
Country of origin	The Netherlands 91.3% Other 8.6%	The Netherlands 88.7% Other 11.3%
Marital status	Cohabiting 51.3% Married 38.7% Single 5.1% Partner, not married 3.9%	Cohabiting 45.2% Married 33.5% Single 13.9% Partner, not married 7.4%
Gender of the child	Male 49.2% Female 50.7% Unknown 0.1%	Male 46.5% Female 53.5%

### Measures

#### Childbirth Experience

Childbirth experience was assessed through five items asking about how women look back on their experiences of childbirth. These items were taken from the ‘Childbirth and breastfeeding’ questionnaire used in the Generations^2^ study, which was based on questions that were used in a national study in the Netherlands on the effects of birth experiences (35). The first item asked ‘How do you look back on your experience of birth?’ and was rated on a 5-point Likert-scale: (1) I am very happy with the way things went (2) I am quite happy with the way things went, (3) I have no special feelings about the birth, (4) I am not quite happy with the way things went, and (5) I am very unhappy with the way things went). The answers were dichotomized to no-risk (answer options 1 to 3) and risk (answer options 4 to 5). The second item asked ‘Which emotions do you have when you look back on your experience of birth?’ giving a list of various positive (11) and negative (13) answer options (e.g., upset, proud, happy, and restless). This item relies on an existing scale on positive and negative affect (36). The frequency of these emotions was rated on a 5-point Likert-scale ranging from (almost) never to very often. Positive items were re-coded so that higher scores refer to higher risk (more negative emotions). After calculating average sum scores, these were dichotomized to no-risk (scores below median, median = 2) and risk (scores above median). The third item asked ‘How did you experience birth?’. Answer options consist of two emotions (i.e., fantastic and awful) that were scored on a 7-point Likert-scale: from very fantastic to not at all fantastic and from very awful to not at all awful. The former emotion (i.e., fantastic) was reversed so that higher scores refer to higher risk (more negative emotions). After calculating an average sum score of these two emotions, these were dichotomized to no-risk (scores below median, median = 2) and risk (scores above median). The fourth item asked ‘Was there a moment during birth you thought that your own life was at risk?’ and the fifth ‘Was there a moment during birth you thought that your baby’s life was at risk?’. Both items were rated either ‘yes’ (risk) or ‘no’ (no-risk). Frequencies of risk and no-risk in each item are presented in **Table 2**. The five items were combined into a latent factor reflecting childbirth experience, of which the validity was tested in the current study.

**Table 2:** Frequencies of negative (‘risk’) experiences in the retrospective birth experience items.

Retrospective birth experience	Frequency of negative experiences
1. How do you look back on your childbirth?	21.1%
2. Which emotions do you have when you think of the birth?	53.2%
3. How did you experience the birth?	46.5%
4. Did you have a moment during the birth, where you thought that your own life was at risk?	9.5%
5. Did you have a moment during the birth, where you thought that your baby’s life was at risk?	29.6%

#### Parenting Stress

Parenting stress was assessed using the parent domain of the Dutch version of the Parenting Stress Index [PSI (37, 38)], which is a validated self-report on stress symptoms related to parenting. The parent domain consists of seven subscales: lack of parenting competence (e.g., ‘When something is wrong with my child, such as illness or hospitalization, I doubt my abilities as a parent’), role restriction (e.g., ‘In order to meet the needs of my child I have to sacrifice more of my life than I expected.’), difficulties with parent-child relationship (e.g., ‘It is rather difficult for me to understand what my child wants or needs.’), depression (e.g., ‘Immediately after the birth of my child I felt more sad and more depressed than I expected.’), social isolation (e.g., ‘I feel alone and without friends.’), health problems (e.g., ‘Because of the family rush I have the feeling I have more physical complaints than before, without children.’), and partner conflict (e.g., ‘The raising of this child has caused more problems in the relationship with my partner than I thought.’). The items are rated on a 6-point Likert-scale ranging from totally disagree (1) to totally agree (6), higher scores referring to higher levels of parenting stress. Internal consistency (Cronbach’s alpha) for the parent domain in the current study was .94 for both mother and partner report. Furthermore, mothers’ and partners’ parenting stress was significantly, although not strongly, correlated at three months postpartum (*r* = .37, *p* <.05).

#### Infant-Parent Attachment

Infant-parent attachment was measured with the Strange Situation Procedure [SSP; (39)], a gold-standard measure of infant-parent attachment (40). The SSP is a structured observational procedure in which parent and child go through eight three-minute episodes (i.e., play, an encounter with a stranger, two separations from the mother, and two reunions with the mother) that prompt the child’s attachment behavior allowing attachment security to be coded. The situations were videotaped, and the two reunion episodes were coded for proximity seeking, contact maintaining, resistant, and avoidant behavior. The Ainsworth et al. (39) coding system was used to classify secure, avoidant, and resistant attachment, and the Main & Solomon (26) coding system to score disorganized attachment. Secure infants were able to maintain proximity and contact with the mother after the reunion and returned to play quickly, while avoidant infants were observed to ignore their mother by focusing on play and resistant infants remained angry at their mother and could not return to play. Disorganized infants, in turn, did not have an organized attachment strategy, and therefore showed disoriented and contradictory behavior during the reunion with the parent. These four categories can be collapsed into secure and insecure (i.e., avoidant, resistant, and disorganized). In addition to this dichotomized classification, a continuous score of attachment disorganization was used to test more specifically the impact of birth experience and parental stress on attachment disorganization. The SSP scoring was done by five reliable and blinded coders, whose agreement on the classifications varied from a kappa of .62 to .91.

### Procedure

Participants were recruited via the study website and midwifery practices in Amsterdam and the surrounding area. Women in the ‘at-risk’ group were additionally recruited from youth care facilities and institutes. Data were collected via questionnaires at 3 months (i.e., birth experience, maternal and paternal PSI) and at 12 months (i.e., maternal PSI) postpartum, as well as during an assessment at the research facility shortly after the child turned one year (i.e., SSP). Participants received the questionnaires and returned them to the research team. If questionnaires were not returned, participants received a reminder email and, if needed, were contacted by phone. All participants signed a written informed consent, and for participants under the age of 18 years, a written informed consent was obtained also from parents and/or other legal guardians.

### Data Analysis

An online power calculator developed specifically for structural equation models (41) was used to calculate the required sample sizes. To find a medium or small effect for the first research question, a sample of 200 participants was needed for the analyses with mothers only, while 400 participants were needed for adequate power with paternal stress included. Adding two demographic variables as possible confounders made these numbers increase to 700 and 1,100, respectively. For the second and third research question, a sample of 100 participants was needed to detect a medium effect and a sample of 947 participants to detect a small effect.

The preliminary data exploration was done using IBM SPSS Statistics (version 26.0). First, Little’s MCAR test was used to analyze the patterns of the missing scores. The test resulted in non-significant *p*-values, which suggests that the missing scores were missing completely at random. Second, the Spearman correlation test was used to investigate whether the five items on women’s birth experiences were related with each other. All items showed significant (*p* <.01), though not strong correlations with the other four items both in the cohort (*r* = 0.18–0.51) and the focus sample (*r* = 0.17–0.45).

Further analyses were conducted with the R package 'lavaan' (42), which is suitable for structural equation modeling. Due to the inclusion of dichotomous variables and the presence of missingness, we used the estimator diagonally weighted least squares (DWLS). Model fit was assessed using robust estimates of the following model fit indices: Comparative fit index (CFI; ≥.95 is good), Tucker-Lewis index (TLI; >.95 is good), root mean square error of approximation (RMSEA; <.06 is good), and standardized root mean square residual (SRMR; ≤.08 is good) (43).

Prior to the main analyses, we tested whether the five dichotomous items on birth experience could be combined into one latent variable. This is an important step in order to estimate whether the five items assess the same underlying construct. In lavaan, the factor loading of the first indicator of a latent variable (i.e., the first birth experience item) is fixed to 1 by default. Factor loadings for the other variables were left free. In addition, residual variances were added automatically to all variables. This analysis was done separately within the cohort and the focus sample (subgroups normative and at-risk).

The first study objective was to investigate whether the latent variable on birth experience was associated with continuous variables on paternal and maternal stress. The latent variable on birth experience resulting from the previous step was used. First, the association between birth experience and maternal stress was assessed. In a second step, paternal stress was added to the model. Paternal stress at 3 months after birth and maternal stress at 12 months after birth had a covariance between them. Finally, residual variances were added automatically. These analyses were performed with the cohort sample. To ease comparability with research questions 2 and 3, the analyses were repeated with the focus sample, although these analyses were underpowered.

The second study objective investigated whether birth experience was indirectly associated with attachment. First, we tested whether the latent variable on birth experience was indirectly associated with the dichotomized (secure-insecure) indicator of child attachment via a latent variable on overall maternal stress, and whether the latent variable on birth experience was directly associated with child attachment. Maternal stress loaded on the variables maternal stress at 3 months after birth and maternal stress at 12 months after birth. Mean maternal stress scores at both time points were almost similar, respectively M = 119.2 (SD = 31.8) at 3 months and M = 119.9 (SD = 30.7) at 12 months. Further, the correlation between maternal stress scores at both time points was very high (*r* = .73). We decided to use the latent variable on maternal stress instead of maternal stress at 3 or 12months in order to resemble more trait-like (i.e., overall) stress instead of state-like (i.e., situation specific) stress. Residual variances were added automatically. This analysis was done with the focus sample, as attachment data were collected for this subgroup only.

Finally, the previous analyses were repeated with a continuous indicator of child attachment (i.e., disorganized attachment). This analysis was also done with the focus sample.

## Results

### The Measure of Birth Experience

**Figure 2:**
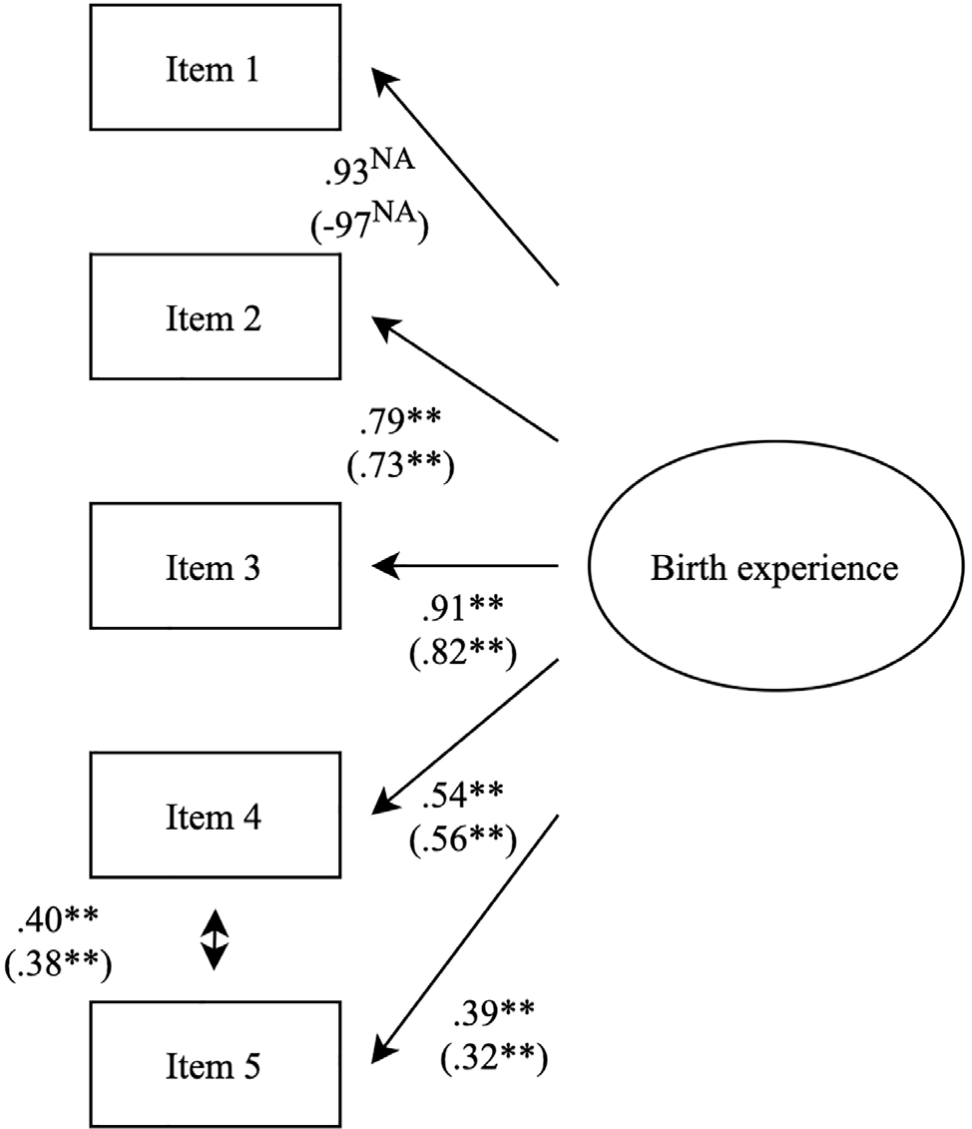
Factor structure for the latent variable on birth experience. Results of the large sample are reported first, and in brackets are results of the smaller sample. These results are of the completely standardized solution, which is why the first birth experience item is not fixed to 1. Significances are marked in the following manner: **p < .05.

First, structural equation modeling was used to test whether the latent variable on birth experience had a good fit in the cohort and the focus sample. The fit was comparable in both samples: CFI was .98 in the cohort and in the focus sample, TLI was .96 in the cohort and .97 in the focus sample, RMSEA was .09 in the cohort and .07 in the focus sample, and SRMR was .07 in the cohort and .07 in the focus sample. Adding a covariance between the thematically linked fourth and fifth items improved model fit. This modification can be explained and understood theoretically, as these two items focus specifically on the fear that a woman may have had for her own or her baby’s life, while the other three items assess more general emotions (both positive and negative) related to the birth experience. Model fit was excellent in both samples: CFI was 1.00 in the cohort and in the focus sample, TLI was 1.00 in the cohort and 1.02 in the focus sample, RMSEA was .00 in the cohort and in the focus sample, and SRMR was .01 in the cohort and .03 in the focus sample. Results are presented in **Figure 2**. Further analyses were conducted with the latent variable combining the five items, as well as a covariance between the fourth and fifth items.

### Birth Experience and Parental Stress

Preliminary analyses indicated that maternal education level and maternal non-Dutch origin were significantly associated with both maternal/paternal stress and the latent variable for birth experience (all *p* <.04), whereas maternal age, marital status, and child gender were not (all *p* >.13). We therefore performed the analyses for the first research question with and without education level and non-Dutch origin as potential confounders in the model. Structural equation modeling was used to test the first study objective: whether birth experience was associated with maternal and paternal stress. Analyses were repeated with four slightly different models (e.g., including or excluding paternal stress and potential confounders) in the cohort sample, which all had good model fit (ranges: CFI = .95–.99, TLI = .93–.99, RMSEA = .04–.06, and SRMR = 0.03–.05. Full results can be found in **Table 3**. In all analyses, mothers’ birth experience was positively associated with maternal stress at 12 months after birth (β ranged from .20 to .22) and paternal stress at 3 months after birth (β = .17).

**Table 3:** Overview of the results for the association between birth experience and parental stress.

		N	Maternal stress		Paternal stress	
			β	p-value	β	p-value
**Large cohort**						
	**Model a**	1252				
	Birth experience		.22	<.001		
	**Model b**	1240				
	Birth experience		.22	<.001		
	Education level		.09	.003		
	Country of origin		-.12	<.001		
	**Model c**	950				
	Birth experience		.21	<.001	.17	<.001
	**Model d**	945*				
	Birth experience		.20	<.001	.17	<.001
	Education level		.09	.003	.11	.001
	Country of origin		-.08	.003	-.08	.006
**Focus cohort**						
	**Model a**					
	Birth experience	222*	.12	.128		
	**Model c**					
	Birth experience	222*	.12	.187	.14	.100

Model a = only maternal stress, no confounders. Model b = only maternal stress, with confounders. Model c = maternal and paternal stress, no confounders. Model d = maternal and paternal stress, with confounders.

*Power was not sufficient in these analyses.

For comparability, the analyses were repeated in the focus sample. Model fit was good for the model with maternal stress only (CFI = .99, TLI = .98, RMSEA = .05, and SRMR = .05) and the model with maternal and paternal stress (CFI = 1.00, TLI = 1.00, and RMSEA = .02, and SRMR = .05). Results showed slightly lower effect sizes for maternal stress (β = .12) and paternal stress (β = .14), which failed to reach significance. However, given that these analyses were underpowered in the focus sample and effects were in the same direction, results remain inconclusive.

### Birth Experience, Maternal Stress, and Child Attachment

For the second study objective, structural equation modeling was used to test whether birth experience was indirectly associated with child attachment via overall maternal stress (i.e., a combination of maternal stress at 3 and 12 months). Additionally, it was tested whether birth experience was directly associated with child attachment. For the first analysis, four-way attachment classifications were dichotomized into secure (51%) and insecure (49%). The underlying distribution of the insecure group was 12% avoidant, 14% resistant, and 23% disorganized. The results showed a good model fit (CFI = 1.00, TLI = .99, RMSEA = .03, and SRMR = .05). Yet, birth experience was neither associated with child attachment indirectly via maternal stress (*β* = .01, *p* = .70) nor directly (*β* = .07, *p* = .49).

For the second analysis, the continuous scale for attachment disorganization was used. The results showed a good model fit (CFI = 1.00, TLI = .99, RMSEA = .03, and SRMR = .05). Similar to the previous analysis, birth experience was neither associated with child attachment indirectly via maternal stress (*β* = .01, *p* = .37) nor directly (*β* = .04, *p* = .67).

## Discussion

This study investigated the impact of mothers’ birth experiences on maternal and paternal stress, as well as the indirect (i.e., via maternal stress) and direct effect of birth experience on parent-child attachment. Our first hypothesis regarding the association between negative birth experience and parenting stress was confirmed. We found that mother’s negative birth experience was indeed positively associated with both the mother’s and partner’s parental stress. However, birth experience was neither associated with child attachment indirectly via maternal stress nor directly.

### Parental Stress

The current findings support previous research by demonstrating a significant effect of mother’s birth experience on parental stress in mothers and their partners (4, 6–9). Importantly, the previous studies including partners have been either qualitative or have included only small samples [e.g., *N* = 56; (7)], which emphasizes the relevance of the current study. Thus, these results show the psychological impact that childbirth can have on the entire family. More precisely, birth and how it is experienced is not only relevant and influential for mothers but can also cause significant parenting stress for partners.

In addition, it is important to note that mothers’ birth experience and paternal stress were assessed 3 months postpartum, while maternal stress was assessed 12 months postpartum. The retrospective childbirth experience reported by mothers was thus not measured at the same time as maternal stress. This strengthens the findings, as the significant association between birth experience and maternal stress cannot be explained by an emotional state that a mother may have experienced when filling out the questionnaires.

Furthermore, mothers’ birth experience at 3 months postpartum affected maternal stress as long as 1 year postpartum. Based on previous research, a traumatic birth experience may negatively impact both mothers’ and their partners’ ability to cope with stress, either directly (4, 31) or by negatively impacting couples’ relationship (31, 44). The latter, in turn, might decrease the amount of support that the partners offer for each other. This may be one of the possible reasons why a negative birth experience can increase partners’ stress and have an impact on mothers’ stress levels even a year after giving birth.

### Child Attachment

The current study did not find a significant indirect association between birth experience and child attachment via maternal stress. Accordingly, the current findings do not support the previous studies that have suggested a link between parental stress and child attachment (20, 21, 23, 27). Furthermore, previous research has reported contradicting findings regarding the possible effect that a negative or traumatic birth experience may have on child attachment (11, 30–32). In the current study, which reflects the largest quantitative study to date with a longitudinal design, we did not find a significant association between a mother’s birth experience and observed child attachment. It has to be noted that this study differs from the previous studies in that it reports on observed parent-child attachment, whereas the previous studies were all performed using either behavioral codings of parental or child behavior or mothers’ self-reports on their feelings and perceptions of the bond with their child. The similarity of these constructs is still up for debate (45). Thus, the question remains: What may protect child attachment from the effects of parental stress and negative birth experience?

One protective factor may be social support. The same way parental stress can make a parent to be less sensitive and responsive to the child (22, 23), social support can help the parent to be more sensitive (46). Sensitive parenting, in turn, is more likely to lead to secure child attachment than insensitive parenting (23, 24). Support may also affect parenting through parental stress, as a lack of partner support may increase stress (21).

Social support and sensitive parenting may similarly explain why we did not find a direct association between a mother’s birth experience and child attachment. As described by family stress theory, support from one’s partner may help a parent to adjust to a stressful situation (i.e., negative birth experience), which, in turn, may lead to more sensitive parenting practices (10). Furthermore, sensitive parenting is more likely to lead to secure child attachment than insensitive parenting (23, 24). In addition, although a child may be a trigger reminding a mother of her traumatic birth experience, some women also express that the direct interaction with their infant, for example, during breastfeeding, helped them to heal mentally from the negative birth experience (33).

Finally, the relatively low power in the analyses in which child attachment was included may explain why we did not find a significant indirect or direct effect of birth experience on child attachment. Collecting observational attachment data requires intensive measures, which makes it difficult to gather large sample sizes. The current sample size would have been large enough to find a medium effect, but not to detect a small effect. The effect sizes were as small as .01 for the indirect effect and .04–.07 for the direct effect. Thus, power was not sufficient to detect this effect. However, it is debatable whether effect sizes this small would have any clinical relevance, should they be significant.

### Strengths, Limitations, and Suggestions for Future Research

The current study has several strengths that ought to be mentioned. First, a longitudinal study design has its benefits over a cross-sectional design, as it may give indications for causal relationships. Second, previous research on birth experience and child attachment have been strongly mother-focused, whereas the current study included also parenting stress data from partners. Third, unlike in a majority of the previous studies, we used a gold standard observational measure to assess child attachment, which adds new information on the attachment relationship to the literature on mother-reported bonding and sensitivity. Finally, in the past years, the number of studies focusing on psychological aspects of childbirth has been increasing and so has the number of available instruments assessing childbirth experiences. However, up to date, there is no gold standard assessment for birth experience (47). In the current study, data on birth experience was collected using a collection of items that were found important, and some of which had been used in previous studies (35, 36). The utilized five items were shown to assess a well-fitting one-factor construct for childbirth experience, both in the cohort and focus sample. It would be interesting to compare outcomes with our instrument to other instruments measuring birth experience, such as the W-DEQ (48) and the CEQ (49), in the future.

However, this study also has limitations that need to be considered when generalizing the findings to other samples. First, although previous studies included women with both negative and traumatic birth experiences, it is possible that, in the current sample, even the most negative birth experiences were not necessarily traumatic. For instance, as can be seen in **Table 2**, only a minority of women had experienced fear for their own or their baby’s life. Thus, it is possible that, for example, in a sample of women with specifically traumatic birth experiences, the indirect and direct effects of birth experience on child attachment may have been larger. Second, although parental stress was assessed in both mothers and their partners, birth experience data was collected only from mothers. Assessing birth experience in partners and mothers would lead to a better understanding of the inter-correlation and effect of both of these. To date, only a limited number of studies have included birth experience data reported by both mothers and their partners [e.g., (5)]. In this context, it would be interesting to measure birth experiences at multiple time points in the first year, ideally starting as soon as possible after delivery, in order to examine whether the reflection on experiences of both partners change over time. Another suggestion for future research would be, instead of investigating new possible mediators in the association between birth experience and child attachment, to assess which factors may moderate the association between birth experience and child attachment (i.e., protective factors). One of the factors of interest would be the moderating effect of partner support, as the current findings hint at a protective effect of partner support in line with family stress theory (10).

## Conclusion

This longitudinal study, combining both self-reported and observational data, as well as multiple informants (i.e., mothers, partners, and observers), investigated the effects that a mother’s birth experience may have on maternal and paternal stress. Furthermore, this study investigated whether birth experience is indirectly associated with child attachment via maternal stress, and whether birth experience is directly associated with child attachment. Both self-reported maternal and paternal parenting stress were found to be associated with a mother’s self-reported birth experience, even until a year after birth. However, self-reported birth experience was neither indirectly nor directly associated with observed infant-parent attachment. These findings emphasize the impact that childbirth may have on both parents, and this way on the family functioning. Thus, after a family has went through a negative birth experience, care professionals ought to support both parents and the couple’s relationship, instead of focusing only on the new mother. Furthermore, the long-lasting effect of a negative birth experience on parental stress emphasizes the need for a strong collaboration between various care sectors that are involved in a family’s life during the first year postpartum (e.g., antenatal care and family counselling). Future research is needed to further investigate which protective factors may weaken the association between birth experience and parental stress, as this will hopefully enable better support for families who have experienced a negative or traumatic childbirth experience.

## Data Availability Statement

The datasets presented in this study can be found in online repositories. The names of the repository/repositories and accession number(s) can be found here: doi: 10.34894/IETFLC.

## Ethics Statement

The studies involving human participants were reviewed and approved by Medical Ethical committee of the VU Medical Centre (METc), registration number NL24319.029.08. Written informed consent to participate in this study was provided by the participants’ legal guardian/next of kin.

## Author Contributions

All authors contributed to the presented idea (i.e., research questions) and the theoretical framework. MV and MO were involved in data collection for the large longitudinal study that offered data for the current study. MV initiated and verified the analytical methods. AH conducted the analyses and all authors discussed the results. AH designed the figures and tables. AH wrote the manuscript with critical feedback and input from MV and MO.

## Funding

This study was supported by a grant from the foundation Stichting tot Steun Netherlands and a grant from the Netherlands Orgqanization for Scientific Research (NWO; grant number 400-09-123).

## Conflict of Interest

The authors declare that the research was conducted in the absence of any commercial or financial relationships that could be construed as a potential conflict of interest.
